# Supplementary material for: Discovery and pharmacophoric characterization of chemokine network inhibitors using phage-display, saturation mutagenesis and computational modelling
Source: Nat Commun. 2023 Sep 16;14:5763. doi: 10.1038/s41467-023-41488-z (PMC10505172; doi:10.1038/s41467-023-41488-z)
Supplement: Supplementary file 4 — Source Data [file 41467_2023_41488_MOESM4_ESM.zip › SourceData/scripts/xngs_20230809084627.tar!/xngs/analysis/SV_EVAA7_filteredheatmap.pdf]

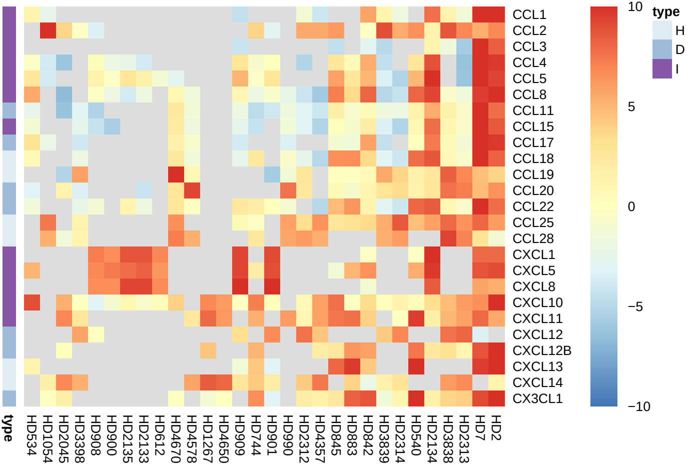

CCL1
CCL2
CCL3
CCL4
CCL5
CCL8
CCL11
CCL15
CCL17
CCL18
CCL19
CCL20
CCL22
CCL25
CCL28
CXCL1
CXCL5
CXCL8
CXCL10
CXCL11
CXCL12
CXCL12B
CXCL13
CXCL14
CX3CL1
type
HD534
HD1054
HD2045
HD3398
HD908
HD900
HD2135
HD2133
HD612
HD4670
HD4578
HD1267
HD4650
HD909
HD744
HD901
HD990
HD2312
HD4357
HD845
HD883
HD842
HD3839
HD2314
HD540
HD2134
HD3838
HD2313
HD7
HD2
10
5
0
−5
−10
type
H
D
I
